# Systemic lupus erythematosus presenting as hyponatremia-associated rhabdomyolysis: A case report

**DOI:** 10.1097/MD.0000000000027390

**Published:** 2021-10-01

**Authors:** In Hee Lee, Seong Cho, Dong Jik Ahn, Min-Kyung Kim

**Affiliations:** a Department of Internal Medicine, Daegu Catholic University School of Medicine, Daegu, Republic of Korea; b Department of Internal Medicine, HANSUNG Union Internal Medicine Clinic and Dialysis Center, Daegu, Republic of Korea; c Department of Pathology, Dongguk University College of Medicine, Gyeongju, Republic of Korea.

**Keywords:** hyponatremia, lupus nephritis, rhabdomyolysis, systemic lupus erythematosus

## Abstract

**Rationale:**

Systemic lupus erythematosus (SLE) is an autoimmune disease that involves multiple organs and causes various clinical manifestations. Cases of rhabdomyolysis as the initial presentation of SLE are rare, and there are no reported cases of SLE presenting hyponatremia-associated rhabdomyolysis as the first manifestation. Herein, we report a case of SLE with lupus nephritis in a patient with acute hyponatremia-associated rhabdomyolysis.

**Patient concerns:**

A 44-year-old woman was admitted with complaints of altered consciousness, myalgia, and red-brownish urine that first appeared three days prior. Peripheral blood tests revealed elevated creatine kinase (19,013 IU/L) and myoglobin (5099 U/L) levels and severe hyponatremia (111 mEq/L) with no azotemia. Urinalysis showed nephritic sediments.

**Diagnosis:**

Whole-body bone scintigraphy showed increased uptake of radiotracer in the both upper and lower extremities. Serological evaluation revealed the presence of anti-nuclear (speckled pattern, 1:640), anti-double stranded DNA, and anti-Smith antibodies and absence of anti-Jo-1 antibody. A kidney biopsy demonstrated mesangial proliferative (class II) lupus nephritis.

**Interventions:**

Fluid therapy, including intravenous administration of 3% NaCl, was initiated. After three consecutive days of intravenous methylprednisolone (1 g/d), oral prednisolone (1 mg/kg/d), mycophenolate mofetil, and hydroxychloroquine were administered.

**Outcomes:**

On day 28, the patient was discharged with marked resolution of SLE-associated symptoms and laboratory findings. Lupus reactivation was not present during the subsequent six-month follow-up.

**Lessons:**

Hyponatremia-associated rhabdomyolysis can be the first manifestation of SLE. Moreover, prompt fluid therapy and timely administration of immunosuppressive agents in SLE patients presenting with hyponatremia and rhabdomyolysis can significantly help alleviate disease activity and improve clinical outcomes.

## 1 Introduction

Systemic lupus erythematosus (SLE) is a chronic multisystem disease that involves multiple organs and causes various clinical manifestations and fatal complications. Common constitutional symptoms include general fatigue, fever, and weight loss. Symptoms of the musculoskeletal and mucocutaneous systems, including myalgia, arthralgia, arthritis, and photosensitive skin lesions such as malar rash are the most common signs of organ involvement.^[1]^ In contrast, acid-base disturbances and electrolyte disorders in patients with SLE are not well known. Renal involvement often causes lupus nephritis (LN) type glomerular disease, and in rare cases, comorbidity with tubulointerstitial nephritis can lead to renal tubular acidosis, hypokalemia, and hyperkalemia.^[2,3]^ Central nervous system (CNS) involvement in SLE can result in hyponatremia (serum sodium [SNa] < 135 mEq/L) associated with syndrome of inappropriate antidiuretic hormone secretion.^[4,5]^ In patients with acute severe hyponatremia (SNa < 115 mEq/L), cerebral edema can occur and lead to neurologic complications such as headache, vomiting, disordered consciousness, convulsions, death, and rhabdomyolysis in rare cases.^[6,7]^

Rhabdomyolysis is a clinical and biochemical syndrome characterized by muscle weakness, myalgia, muscular swelling, and myoglobinuria due to acute injury of the skeletal muscles arising from diverse causes.^[8]^ Cases of rhabdomyolysis in patients with preexisting SLE have been reported,^[9–16]^ but rhabdomyolysis as the first clinical symptom of SLE is rare.^[17]^ There are no reported cases of SLE presenting hyponatremia-associated rhabdomyolysis as the first manifestation in patients with no specific history. Here, we review the literature and report acute hyponatremia-associated rhabdomyolysis in a patient diagnosed with SLE and class II LN.

## 2 Case report

A 44-year-old woman visited our emergency department with complaints of altered consciousness and generalized myalgia that first appeared three days prior. The patient had intermittent low-grade fever, general weakness, and decreased food intake for the past 10 days due to nausea, vomiting, and loss of appetite. At admission, her blood pressure (120/90 mm Hg), heart rate (80 beats/min), respiration rate (20 breaths/min), and body temperature (36.8°C) were assessed. She was diagnosed with hypothyroidism 10 years prior to admission but did not have diabetes mellitus, hypertension, tuberculosis, or kidney disease. The patient was taking levothyroxine (88 μg) and calcium carbonate (1,250 mg) daily with no history of other specific medications. There were no recent episodes of trauma, seizures, tremors, or muscle stiffness. While the oral mucosa was not dry, the patient had decreased skin turgor. The patient was lethargic but showed normal pupillary reflexes. No localized neurological deficits were observed. The Glasgow Coma Scale score was 13 points (E3M6V4). Peripheral blood examinations performed at admission showed mild leukocytosis, hypoalbuminemia, elevated levels of muscle enzymes creatine kinase (CK) and lactate dehydrogenase (LDH), and severe hyponatremia (111 mEq/L). Azotemia was not present (Tables 1 and 2). Urine was grossly dark-brown in color, and routine urinalysis confirmed hematuria (dysmorphic red blood cells [RBCs] > 90%), leukocyturia, non-nephrotic albuminuria, RBC casts, and myoglobinuria (Table 1). Blood coagulation profiles (prothrombin time, activated partial thromboplastin time, fibrinogen levels) were all within normal ranges. Venereal disease tests, viral serologies (influenza A/B, hepatitis B surface antigen, hepatitis C antibody, and anti-HIV antibody), and SARS-CoV-2 PCR test from a nasopharyngeal swab were all negative. The fractional excretion of filtered sodium (FE_Na_), urea (FE_Urea_), and uric acid (FE_UA_) in spot urine were 0.03%, 7.1%, and 2.69%, respectively. Brain magnetic resonance imaging revealed no abnormal findings. Based on these findings, the patient was suspected to have acute hypovolemic hyponatremia, hyponatremic encephalopathy, and rhabdomyolysis.

**Table 1:** Laboratory findings on admission.

Variable	Patient's value	Reference	Variable	Patient's value	Reference
Blood cell count			Urinalysis		
White blood cell (/μL)	10,600	3,600–9,600	Albumin	4+	Negative
Hemoglobin (g/dL)	14.7	12.9–16.9	Occult blood	3+	Negative
Platelet (×10^3^/μL)	229	140–380	RBC (/HPF)^∗^	10–29	0–1
ESR (mm/hour)	20	0–10	WBC (/HPF)	10–29	0–3
Serum biochemistry			RBC cast	2+	None
Albumin (g/dL)	2.5	3.5–5.1	Urine chemistry		
Blood urea nitrogen (mg/dL)	18.5	8–19	Sodium (mEq/L)	10	40–220
Creatinine (mg/dL)	0.5	0.6–1.2	Osmolality (mOsm/kg)	775	300–900
Sodium (mEq/L)	111	135–148	Protein-to-Cr (g/g)	1.62	<0.2
Total CO_2_ (mEq/L)	19.6	22–28	Serology		
Calcium (mg/dL)	7.8	8.2–10.2	IgG (mg/dL)	1,806	700–1,600
Phosphorus (mg/dL)	3.3	2.5–4.5	IgA (mg/dL)	272.9	70–400
Uric acid (mg/dL)	3.7	2.4–5.7	IgM (mg/dL)	103.5	40–230
Osmolality (mOsm/kg)	237	285–294	C3 (mg/dL)	29.4	90–180
C-reactive protein (mg/L)	24.9	<5	C4 (mg/dL)	10.2	10–40

C = serum complement, Cr = creatinine, ESR = erythrocyte sedimentation rate, HPF = high power field, Ig = immunoglobulin, RBC = red blood cell, WBC = white blood cell

∗ dysmorphic > 80%.

**Table 2:** Evolution of serum parameters during hospitalization.

Time (HD/hours)	HD 1	HD 2		HD 3	HD 5^∗^	HD 7	HD 10^†^	HD 14	HD 28	Reference
	0	6	12	36	84	132	204	300	636	
Sodium (mEq/L)	111	114	114	127	138	131	136	134	135	135–148
CK (IU/L)	19,013	19,129	17,765	25,512	60,092	39,303	10,955	802	380	30–180
LDH (IU/L)	1,679	1,587	1,612	1,625	2,064	1,693	1,714	792	146	<250
AST (IU/L)	742		725	850	1,733	1,472	886	105	15	<35
ALT (IU/L)	298		295	264	411	461	504	236	43	<40
Myoglobin (U/L)			5,099	17,482	15,883	8,735	1,049	147	44	25–58

ALT = alanine aminotransferase, AST = aspartate aminotransferase, CK = creatine kinase, HD = day of hospitalization, LDH = lactate dehydrogenase.

∗ whole-body bone scintigraphy.

† kidney biopsy.

A bolus of 100 mL 3% NaCl (1.5 mL/kg) was administered twice for 20 min at 2 h intervals. Subsequently, fluid therapy was maintained via continuous intravenous (IV) administration of 0.9% NaCl at a rate of 150–250 mL/h, and IV administration of ceftriaxone (2.0 g/day) was prescribed for the treatment of a suspected urinary tract infection. The serum sodium concentration gradually increased after fluid and electrolyte supplementation but the muscle aches persisted. Serum levels of muscle enzymes and myoglobin were higher than those measured at admission (Table 2). Despite treatment with antipyretics, several episodes of intermittent high fever (body temperature  > 38.3°C) occurred daily.

**Figure 1:**
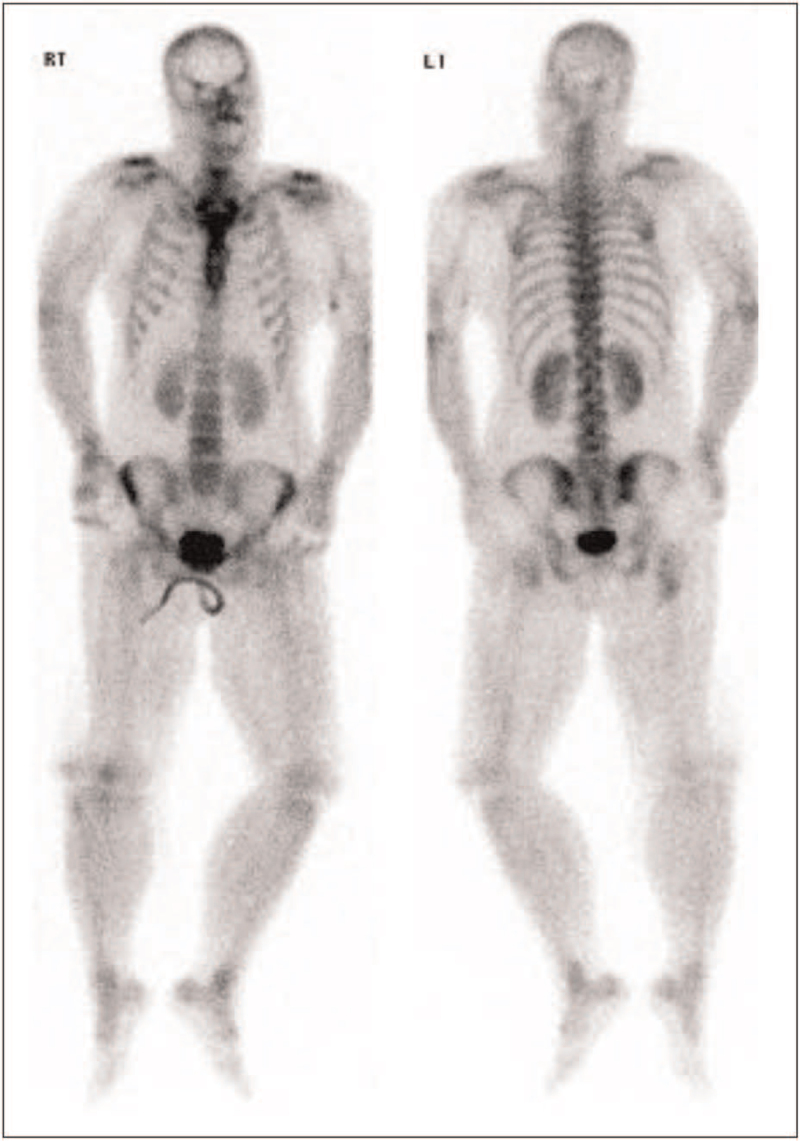
A ^99m^Tc-HDP whole-body bone scintigraphy on the 5th hospital day showed diffusely increased soft tissue uptake of radiotracer in the both upper and lower extremities.

When the patient regained consciousness on day five of hospitalization, a ^99m^Tc-HDP whole-body bone scintigraphy was performed and showed increased soft tissue uptake of radiotracer in the both upper and lower extremities (Fig. 1). Urine and blood cultures revealed no microbial infection. Serological assessment for glomerular disease showed the presence of anti-nuclear antibodies (speckled pattern, 1:640) along with elevated serum immunoglobulin (Ig) G and decreased serum complement (C) 3 (Tables 1 and 3). Urinary protein excretion and creatinine (Cr) clearance in a 24-h urine study were 0.978 g/d and 113.7 mL/min/1.73 m^2^, respectively.

**Table 3:** Initial findings of clinical chemistry and immunology.

Variable	Patient's value	Reference	Variable	Patient's value	Reference
Autoimmune panel			Endocrinology		
ANAs (titer)^∗^	1:640^†^	Negative	Free T3 (pg/mL)	0.983	2.0–4.4
Anti-ds DNA Ab IgG (IU/mL)^∗^	46	<10	Free T4 (ng/dL)	1.13	0.8–1.9
Anti-RNP Ab (U/mL)^∗^	>99,999	<5	TSH (μIU/mL)	1	0.4–4.7
Anti-Sm Ab (U/mL)^∗^	>480	<7	Cortisol (μg/dL)	33.8	4.82–19.5
Anti-Ro/SS-A Ab (U/mL)^∗^	>240	<7	ACTH (pg/mL)	21.7	4.7–48.8
Anti-La/SS-B Ab (U/mL)^∗^	>320	<7	PRA (ng/mL/hour)	47.04	0.32–1.84
Anti-histone Ab (U)^∗^	2.7	<1.0	Aldosterone (ng/dL)	49.9	1.76–23.2
Anti-Jo-1 Ab (U/mL)	<0.01	<7	ADH (pg/mL)	24.91	<14.04

Ab = antibody, ACTH = adrenocorticotrophic hormone, ADH = antidiuretic hormone, ANAs = anti-nuclear antibodies, ds = double stranded, PRA = plasma renin activity, TSH = thyroid stimulating hormone.

∗ seropositive.

† speckled pattern.

A peripheral blood test performed on day seven showed white blood cell counts of 1,700/μL (neutrophils 81.9%), hemoglobin of 7.6 g/dL, and platelet counts of 72,000/μL. Serological screening for autoimmune disease revealed the presence of all autoantibodies except anti-Jo-1 antibody (Table 3). Consequently, the patient was diagnosed with SLE based on the Systemic Lupus International Collaborating Clinics classification criteria.^[18]^ The calculated SLE disease activity index (SLEDAI) was 23 points after excluding scores corresponding to myositis, which suggested high immunologic activity.^[19]^ Accordingly, IV methylprednisolone (1 g/day) was administered for three consecutive days, followed by a switch to oral prednisolone (60 mg; 1 mg/kg/d).

**Figure 2:**
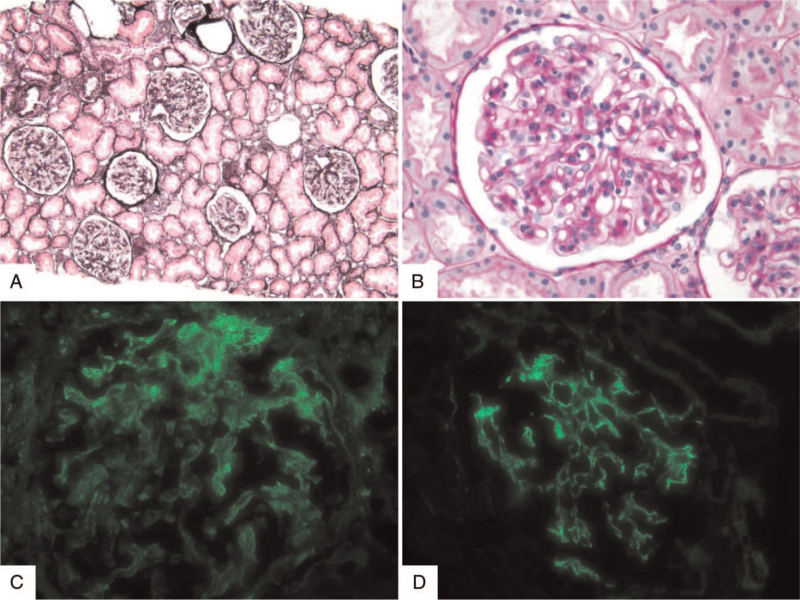
Microscopic features of renal biopsy. (A) Light microscopy shows no evidence of glomeruli with segmental sclerosing or necrotizing lesions (methenamine silver stain, ×100). (B) Mesangial hypercellularity is present with matrix expansion (B: periodic acid Schiff, ×400). (C) Immunofluorescence shows immunoglobulin (Ig) deposits in the mesangium (anti-IgG immunofluorescence, ×400). (D) Mesangial deposition of complement (C) 3 shows a similar pattern of deposition of IgG (anti-C3 immunofluorescence, ×400). C = complement, Ig = immunoglobulin.

**Figure 3:**
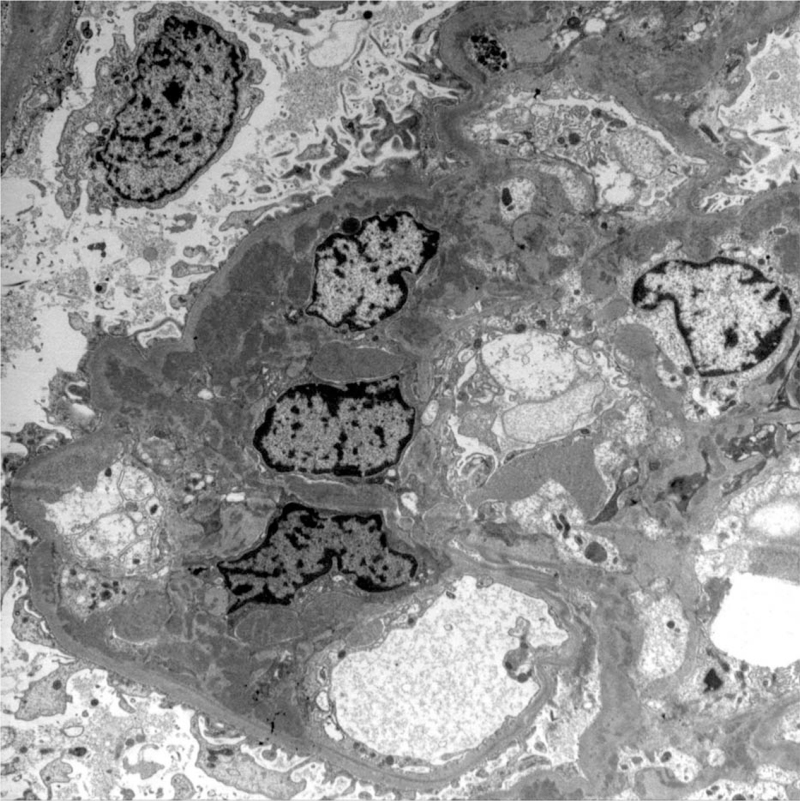
Electron microscopy demonstrates subendothelial electron-dense deposits in addition to the presence of abundant mesangial deposits (transmission electron microscopy, ×3500).

On day 10 of hospitalization, blood urea nitrogen and serum Cr levels were 23.7 and 1.2 mg/dL, respectively. A percutaneous kidney biopsy was performed to assess renal lesions associated with SLE. Light microscopic examination of the kidney specimen revealed 38 glomeruli, of which two exhibited segmental glomerulosclerosis. There were no other significant pathologies (crescent formation, tubulointerstitial fibrosis), inflammatory cell infiltration, or proliferation (Fig. 2A, B). Immunofluorescence microscopy showed IgG, IgA, C3, C1q, kappa, and lambda fluorescence depositions in the glomerular basement membrane and mesangium (Fig. 2C, D). Electron microscopy revealed local loss of foot processes and electron-dense deposits in the subendothelial regions and mesangium (Fig. 3). The patient was diagnosed with mesangial proliferative (class II) LN based on the microscopic findings. After renal biopsy confirmation, oral administration of mycophenolate mofetil (2 g/d) and hydroxychloroquine (200 mg/d) was included. Oral prednisolone was tapered each fortnight. After initiating combined immunosuppressive therapy, a gradual improvement in clinical symptoms (fever, generalized ache) and laboratory findings (muscle enzyme levels, urinalysis results) occurred.

On day 28 of hospitalization, anti-double stranded DNA antibody and serum C3 levels were 15 IU/mL (reference: <10 IU/mL) and 71.3 mg/dL, respectively, with a significant improvement from levels at the time of admission. Accordingly, the patient was discharged. At 6 months after hospital discharge, the patient showed serum Cr of 0.3 mg/dL, spot urine protein-to-Cr of 0.133 g/g, RBC 1–4/high-power field, and white blood cell 1–3/ high-power field. Currently, the patient is under follow-up observation with maintenance of oral medications (10 mg/day prednisolone, 200 mg/day hydroxychloroquine).

## 3 Discussion

SLE, characterized by repeated recurrence and remission of various inflammatory lesions, is a chronic disease of the connective tissue caused by abnormalities in the immune system. SLE may involve major organs, including the respiratory, cardiovascular, renal, and CNS systems, to cause life-threatening complications.^[1]^ Among the comorbid kidney diseases in approximately 50% of SLE patients, LN is the most commonly reported. Water-electrolyte imbalance disorders, such as loss of urine concentrating ability, hypernatremia, abnormal serum calcium concentration, and acid-base disturbances, are rare and are significantly associated with tubulointerstitial inflammation that occurs alone or in combination with LN (mostly class IV LN).^[2,3]^ When chronic inflammatory lesions, such as chronic tubulointerstitial nephritis, become obvious in SLE patients, renal excretion of sodium through damaged tubules may increase significantly.^[20]^ Administration of drugs, such as cyclophosphamide, non-steroidal anti-inflammatory drugs, and diuretics, is also associated with a decrease in serum sodium levels in patients with SLE. Given the diversity of hyponatremia causes, the clinical significance and frequency of hyponatremia in SLE patients have not been established. Recent studies have reported that hyponatremia in SLE patients is directly proportional to serum albumin, C3, and serum chloride levels and is inversely correlated with inflammatory parameters, such as SLEDAI, C-reactive protein (CRP), and erythrocyte sedimentation rate.^[21–23]^ In patients with connective tissue disease such as SLE, pre-inflammatory cytokine levels, including interleukin (IL)-1 beta and IL-6, may be increased to promote antidiuretic hormone secretion, which could lead to hyponatremia.^[24]^ In our case, the patient was suspected of hyponatremic encephalopathy at admission as signs of systemic disease such as liver cirrhosis, congestive heart failure, nephrotic syndrome, or renal failure were absent. CNS lupus and neuropsychiatric disorders were also excluded based on neurological examination and brain magnetic resonance imaging. Moreover, there were no endocrine disorders, malignancies, pulmonary diseases, or medications associated with hyponatremia. Physical examination and laboratory findings (urine sodium concentration < 10 mEq/L, urine osmolality 775 mOsm/kg, fractional excretion of major solutes FE_Na_, FE_Urea_, and FE_UA_) suggested that a decrease in extracellular fluid due to a decrease in dietary intake and extrarenal sodium loss were more likely than syndrome of inappropriate antidiuretic hormone secretion.^[25]^ The elevated antidiuretic hormone level (24.91 pg/mL) was suspected to contribute to the development of osmoregulation for hypovolemia. In this patient, hypoalbuminemia, decreased C3, increased CRP, and high SLEDAI implied an increase in lupus activity, but the significant association between these findings and the occurrence of severe hyponatremia was inconclusive.

Diagnosis of rhabdomyolysis was possible based on the presence of myoglobinuria, elevated serum myoglobin, and increased serum CK concentration that exceeded normal levels by five-fold following injury to the skeletal muscles, causing the release of intracellular contents into the blood stream.^[8]^ Rhabdomyolysis is usually caused by trauma, excessive alcohol intake, convulsions, drugs, strenuous exercise, heat stroke, and infections. In rare cases, rhabdomyolysis is caused by electrolyte disturbances, including hypokalemia, hypocalcemia, hypophosphatemia, and hyponatremia.^[8]^ Few hypotheses have been proposed to explain the pathogenesis of hyponatremia-associated rhabdomyolysis.^[7]^ When extracellular fluid osmolality decreases due to hyponatremia, cells expand, and intracellular potassium is continuously released into the extracellular compartment and cellular transmembrane potential decreases in the potassium-deficient myocytes leading to muscle destruction.^[7]^ Another hypothesis points to abnormalities in the Na^+^/Ca^++^ exchange pump in the cell membrane. As sodium concentration in the extracellular fluid decreases, the Ca^++^ output, which is linked to the Na^+^ input, also decreases. As a result, sodium accumulates inside cells and activates enzymes (neural proteases and lipases) that cause myolysis.^[26]^ The rate of serum sodium concentration decline and hyponatremia severity are significantly associated with the degree of muscle injury. Also, the failure to regulate cell volume during rapid correction of hyponatremia can cause increased cell membrane fragility and leakage of intracellular enzymes.^[27]^ In such cases, blood CK levels are markedly increased 48–96 h after hyponatremia onset. Myalgia, muscle swelling, and increased muscle enzyme levels that occur after rhabdomyolysis development are similar in inflammatory myopathy associated with immune disorders. Therefore, when the cause of rhabdomyolysis is not clear, autoimmune diseases should be excluded via autoantibody screening tests.^[17,28]^

To date, there are only nine English-written articles reporting on SLE patients with rhabdomyolysis since the first reported case in 1994 (Table 4). The mean age at the time of diagnosis was 35 (range: 25–45) years, and all patients were women. Each patient exhibited different features with respect to the primary causes of rhabdomyolysis, including medications (cyclosporine, atorvastatin, and quinacrine) and infectious diseases (salmonellosis and dengue fever). In a 28-year-old woman with initial SLE presentation diagnosed with rhabdomyolysis, the primary causes of rhabdomyolysis were strenuous exercise and oral contraceptive use.^[17]^ The mean peak serum Cr concentration during rhabdomyolysis was 2.4 (range: 0.91–4.9) mg/dL, and four patients (44.4%) showed acute kidney injury (serum Cr > 2.0 mg/dL). The peak serum CK level (range: 1,846–304,700 IU/L) and length of hospital stay (range: 0–60 days) also varied from case to case. The patient in our case was a 44-year-old woman with peak serum Cr and CK levels during her 28 days of hospital stay at 1.2 mg/dL and 60,092 IU/L, respectively, with no clinical evidence implicating viral disease, bacterial infection, or medication. Polymyositis, which is considered an alternative cause of rhabdomyolysis, was ruled out based on the absence of anti-Jo-1 antibody. Hypothyroidism-associated rhabdomyolysis was also excluded based on hormone test results (Table 2). The patient was diagnosed with acute hyponatremia-associated rhabdomyolysis before the confirmation of SLE. The patient showed a gradual increase of muscle enzyme levels in serum, including CK and LDH, after admission, and the levels peaked on day five of hospitalization. This pattern was similar to that observed in previously reported cases of hyponatremia-associated rhabdomyolysis.^[7,27]^ Immunosuppressive therapy, initiated on day seven of hospitalization including IV administration of corticosteroid, was postulated to significantly improve both SLE-specific symptoms and rhabdomyolysis.

**Table 4:** Cases of rhabdomyolysis in patients with systemic lupus erythematosus.

No	Year reported	Age (yr)	Sex	Peak Cr (mg/dL)	Peak CK (IU/L)	Duration^∗^ (yr)	Etiology	Comorbidity	HP (d)	Clinical outcome
1	1994^[9]^	39	F	NR	17,260	12	Discoid lupus	Polymyositis	60	Remission
2	1999^[10]^	40	F	2.2	1,846	23	Atorvastatin, Cyclosporine	Kidney transplantation	4	Remission
3	2000^[11]^	27	F	1.39	45,429	3	*S. enteritidis* bacteremia	Acute cholecystitis	34	Remission
4	2005^[12]^	45	F	NR^‡^	39,000	NR	Quinacrine	Dystrophic calcinosis	NR	Remission
5	2011^[13]^	36	F	4.9	75,000	13	Unspecific myositis^§^	Pulmonary infection	10	Death
6	2014^[14]^	39	F	0.91	45,265	4	Dengue fever	Compartment syndrome	34	Remission
7	2018^[15]^	36	F	3.59	304,700	5	Gastroenteritis	None	NR	Remission
8^†^	2019^[17]^	28	F	1.2	13,776	0	Exercise, Oral contraceptive	Chronic azotemia	0	Remission
9	2020^[16]^	25	F	2.8	13,585	NR	Fungal infection	Mesenteric panniculitis^§^	NR	Death
10^†^	Present	44	F	1.0	60,092	0	Acute hyponatremia	Hypothyroidism	28	Remission

CK = creatine kinase, Cr = serum creatinine, d = days, F = female, HP = hospitalization period, No = number, NR = not reported, *S* = Salmonella, yr = years.

∗ Duration of systemic lupus erythematosus prior to occurrence of rhabdomyolysis.

† Initial presentation of systemic lupus erythematosus

‡ Azotemia.

§ Autopsy.

Given the insufficient number of relevant cases, the prognosis of SLE patients with rhabdomyolysis has not yet been analyzed. However, considering the clinical evolution of previous cases, patients with SLE complicated with rhabdomyolysis may have favorable prognoses (Table 4). Early mortality has been documented in SLE patients with rhabdomyolysis due to pulmonary infection caused by unidentified pathogens and bacteremia with invasive fungal co-infection.^[13,16]^ The presence of bacterial or fungal infection should be assessed first in these patients and preemptive antimicrobial therapy should be actively considered when necessary. While the administration of empirical antibiotics was initiated for suspicion of a urinary tract infection, the urine and blood culture results were negative in our patient. Upon admission, our patient presented with hyponatremic encephalopathy, increased levels of CRP and erythrocyte sedimentation rate, and hypoalbuminemia. However, she did not have poor prognostic factors of rhabdomyolysis, namely, azotemia, hypocalcemia, hyperphosphatemia, or metabolic acidosis.^[29]^ By initiating a standardized therapy, including IV fluid therapy and administration of high-dose corticosteroid, our patient did not exhibit exacerbation of SLE or serious complications associated with rhabdomyolysis during hospitalization. Moreover, during the subsequent six-month follow-up, she showed a relatively favorable outcome with no clinical evidence of lupus reactivation.

In summary, we describe a case of a 44-year-old woman with acute hyponatremia-associated rhabdomyolysis that was treated after diagnosis with comorbid SLE and class II LN based on serological evaluation and kidney biopsy. Although the clinical manifestations of SLE may vary significantly, hypovolemic hyponatremia with rhabdomyolysis could also be its initial presentation. Moreover, in SLE patients with acute hyponatremia-associated rhabdomyolysis, prompt fluid therapy combined with timely administration of immunosuppressive agents might help alleviate disease activity and improve clinical outcomes.

## Author contributions

Conceptualization: In Hee Lee.

Data curation: Seong Cho, Min-Kyung Kim.

Formal analysis: Dong Jik Ahn, In Hee Lee.

Methodology: In Hee Lee.

Validation: Dong Jik Ahn, In Hee Lee.

Writing - original draft: In Hee Lee.

Writing - review & editing: In Hee Lee.

## References

[1] Dall’Era M Wofsky D. Firestein GS. Clinical features of systemic lupus erythematosus. Kelly and Firestein's Textbook of Rheumatology. 10th ed. Philadelphia: Elsevier; 2017. 1345–67.

[2] Mori Y Kishimoto N Yamahara H. Predominat tubulointerstitial nephritis in a patient with systemic lupus nephritis. Clin Exp Nephrol 2005;9:79–84. 15830279 10.1007/s10157-004-0338-3

[3] Oliva-Damaso N Oliva-Damaso E Payan J. Acute and chronic tubulointerstitial nephritis of rheumatic causes. Rheuma Dis Clin North Am 2018;44:619–33. 10.1016/j.rdc.2018.06.009 30274627

[4] Brey RL. Neuropsychiatric lupus: clinical and imaging aspects. Bull NYU Hosp Jt Dis 2007;65:194–9. 17922669

[5] Hara H Hasegawa H Iwanaga M. A case of the syndrome of inappropriate secretion of antidiuretic hormone (SIADH) associated with lupus erythematosus in the central nervous system. CEN Case Rep 2013;2:17–22. 28509213 10.1007/s13730-012-0031-4 PMC5413721

[6] Hoorn EJ Zietse R. Diagnosis and treatment of hyponatremia: compilation of the guidelines. J Am Soc Nephrol 2017;28:1340–9. 28174217 10.1681/ASN.2016101139 PMC5407738

[7] Trimarchi H Gonzalez J Olivero J. Hyponatremia-associated rhabdomyolysis. Nephron 1999;82:274–7. 10396001 10.1159/000045413

[8] Cabral BMI Edding SN Portocarrero JP Lerma EV. Rhabdomyolysis. Dis Mon 2020;66:101015. 32532456 10.1016/j.disamonth.2020.101015

[9] Menon S Round JM Isenberg DA. Rhabdomyolysis in a patient with discoid lupus erythematosus. J Rheumatol 1994;21:1967–9. 7837170

[10] Maltz HC Balog DL Cheigh JS. Rhabdomyolysis associated with concomitant use of atorvastatin and cyclosporine. Ann Pharmacother 1999;33:1176–9. 10573315 10.1345/aph.19039

[11] Blaauw AA Tobé TJ Derksen RW Bijlsma JW. A patient with systemic lupus erythematosus and salmonella enteritidis bacteraemia complicated by rhabdomyolysis and acute cholecystitis. Rheumatology (Oxford) 2000;39:110–2. 10662885 10.1093/rheumatology/39.1.110

[12] Creel N Werth V. Rhabdomyolysis associated with quinacrine therapy in a patient with chronic cutaneous lupus erythematosus. J Drugs Dermatol 2005;4:225–7. 15776783

[13] de Carvalho JF da Mota LM Bonfa E. Fatal rhabdomyolysis in systemic lupus erythematosus. Rheumatol Int 2011;31:1243–5. 21127876 10.1007/s00296-010-1674-0

[14] Verdolin LD Borner AR Mussi H Gismondi RA Schau B Ramos RC. Rhabdomyolysis associated with dengue fever in a lupic patient. Rev Bras Reumatol 2014;54:318–21. 25627228 10.1016/j.rbr.2013.02.003

[15] Nguyen D Alsaati F Deitrick J Azhar K Sbar E. Rhabdomyolysis secondary to systemic lupus erythematosus. Auto Immun Highlights 2018;9:05. 10.1007/s13317-018-0105-1 PMC5886906 29623452

[16] Gupta K Kapatia G Rathi M Mitra S Singhal M Sharma N. Mesenteric panniculitis and rhabdomyolysis complicated by invasive fungal co-infection in a case of systemic lupus erythematosus: an autopsy report. Indian J Nephrol 2020;30:329–33. 33707821 10.4103/ijn.IJN_296_19 PMC7869644

[17] Saxena G Mahdi A. Rhabdomyolysis as an initial presentation of systemic lupus erythematosus: a case report. Int J Emerg Med 2019;12:33. 31703554 10.1186/s12245-019-0251-x PMC6842242

[18] Petri M Orbai AM Alarcón GS. Derivation and validation of the systemic lupus international collaborating clinics classification criteria for systemic lupus erythematosus. Arthritis Rheum 2012;64:2677–86. 22553077 10.1002/art.34473 PMC3409311

[19] Gladman DD Ibañez D Urowitz MB. Systemic lupus erythematosus disease activity index 2000. J Rheumatol 2002;29:288–91. 11838846

[20] Eisenhut M. Changes in renal sodium transport during a systemic inflammatory response. Pediatr Nephrol 2006;21:1487–8. 16897000 10.1007/s00467-006-0199-y

[21] Shin JI Park SJ Suh CH. Hyponatremia in patients with systemic lupus erythematosus. Sci Rep 2016;6:25566. 27193532 10.1038/srep25566 PMC4872139

[22] El-Badawy MA El-Mahdi AR El-Sherbiny DA Bawady SAH. Hyponatremia in systemic lupus erythematosus patients: relation to disease activity and fatigue. Egypt Rheumatol 2019;41:283–7.

[23] Yamany A Behiry ME Ahmed SA. Hyponatremia as an inflammatory marker of lupus activity is a fact or fad: a cross-sectional study. Open Access Rheumatol 2020;12:29–34. 32210647 10.2147/OARRR.S237168 PMC7075429

[24] Swart RM Hoorn EJ Betjes MG Zietse R. Hyponatremia and inflammation: the emerging role of interleukin-6 in osmoregulation. Nephron Physiol 2011;118:45–51. 21196778 10.1159/000322238

[25] Decaux G Musch W. Clinical laboratory evaluation of the syndrome of inappropriate secretion of antidiuretic hormone. Clin J Am Soc Nephrol 2008;3:1175–84. 18434618 10.2215/CJN.04431007

[26] Ting JY. Rhabdomyolysis and polydipsic hyponatraemia. Emerg Med J 2001;18:250. 11696527 10.1136/emj.18.6.520 PMC1725741

[27] Morita S Inokuchi S Yamamoto R. Risk factors for rhabdomyolysis in self-induced water intoxication (SIWI) patients. Emerg Med J 2010. 293–6. 10.1016/j.jemermed.2007.09.040 18439783

[28] Mai H Zhao Y Salerno S Li Y Yang L Fu P. Rhabdomyolysis-induced acute kidney injury in a patient with undifferentiated connective tissue disease. Medicine 2019;98:e16492. 31348259 10.1097/MD.0000000000016492 PMC6709088

[29] McMahon GM Zeng X Waikaer SS. A risk prediction score for kidney failure or mortality in rhabdomyolysis. JAMA Intern Med 2013;173:1821–8. 24000014 10.1001/jamainternmed.2013.9774 PMC5152583

